# Influence of anxiety on university students’ academic involution behavior during COVID-19 pandemic: Mediating effect of cognitive closure needs

**DOI:** 10.3389/fpsyg.2022.1005708

**Published:** 2022-09-29

**Authors:** Dongdong Yan, Huanzhe Zhang, Shili Guo, Wen Zeng

**Affiliations:** ^1^Department of Sociology, School of Ethnology and Sociology, Inner Mongolia University, Hohhot, China; ^2^Department of Sociology and Psychology, School of Public Administration, Sichuan University, Chengdu, Sichuan, China; ^3^Institute of Population, Resource and Environmental Economics, School of Economics, Southwestern University of Finance and Economics, Chengdu, Sichuan, China

**Keywords:** COVID-19, perceived risk, anxiety, stress, need for cognitive closure, academic involution behavior

## Abstract

The COVID-19 pandemic has had a significant negative effect on university students’ mental health worldwide. The pandemic has resulted in individuals experiencing increased levels of anxiety and stress as well as intensified concerns about the future due to a rise in uncertainty. To eliminate the anxiety and stress caused by uncertainty, individuals who have high cognitive closure needs are strongly motivated to achieve certainty and seek answers, even if the decisions they make in the process are inappropriate or even irrational. This study attempts to analyze the influence of anxiety and stress on university students’ academic involution behavior during the COVID-19 pandemic through the mediating effect of the need for cognitive closure. Analyzing the survey data collected from 402 university students from 3 different universities through the mediating effect model and path analysis with latent variable (PA-LV), our results indicate that: (1) The COVID-19 pandemic has significantly increased the levels of anxiety and stress experienced by university students. The path coefficient of the perceived risk of contracting COVID-19 to perceived emotions (including anxiety and stress) was 0.352 (*p* < 0.01), (2) anxiety and stress significantly and positively affect academic involution behavior. For each unit of increase in the scores of anxiety and stress, the scores of academic involution behavior increased by 0.408 (*p* < 0.01) and 0.398 (*p* < 0.01) units, respectively, and (3) The need for cognitive closure had a complete and partial mediating effect on the relationship between the perceived risk of contracting COVID-19 and academic involution behavior, as well as anxiety and academic involution behavior, respectively. The results of PA-LV showed that the mediating effect values were 0.106 and 0.044, respectively. The impact of the COVID-19 pandemic has not yet come to an end and so clarifying the relationship between anxiety and academic involution behavior will assist university students to optimize the relationship in their own subjective situations, to establish good learning habits, and reduce psychological distress.

## Introduction

In March 2020, the outbreak of COVID-19 was declared a pandemic by the World Health Organization. The COVID-19 pandemic has had a significant impact on the psychology of the public and the increase in risks caused by the pandemic has resulted in a surge in global widespread anxiety (Qiu et al., 2020; Rajkumar, 2020). University students, with their rich array of emotions, are one of the population subgroups that are more likely than others to experience psychological difficulties (Li et al., 2020). When suddenly faced with the unpredictable nature of the COVID-19 pandemic, university students are prone to experience negative emotional effects, such as stress, panic, anxiety, and depression (Wang et al., 2020). With the continued impact of the COVID-19 pandemic still evident in the second half of 2020, an initially obscure sociological concept known as “involution” emerged among university students and triggered heated discussion and strong reactions. The concept of involution can be traced back to 1963, when Geertz defined it as “these culture patterns which, after having reached what would seem to be a definitive form, nonetheless fail either to stabilize or transform themselves into a new pattern but rather continue to develop by becoming internally more complicated” (Geertz, 1963, p. 81). Huang Zongzhi introduced this concept into economics and called it “growth without development,” that is, “involution growth” (Liu and Qiu, 2004). With the development of society, the meaning of involution is often used to refer to fierce competition, which conveys the anxiety in the culture of high-pressure competition.

When involution is used to explain the current problems being experienced in China’s education system, the difficulties are vividly described as a dilemma of high cost and internal friction without the corresponding return, with typical problems such as educational anxiety, fierce competition, and excessive education (Chen and Bao, 2022; Yang et al., 2022). When it comes to university students, “academic involution behavior” has a new connotation: it refers to a learning behavior mode of increasing negativity, excessive competition, and low productivity that university students adopt in an irrationally competitive environment when faced with learning difficulties (Zheng et al., 2022). Upon investigating the concept, we see that academic involution is not only a problem of excessive competition but also a social crisis of inefficient competition and ineffective development (Yang et al., 2022). Guo (2021) stated that the academic involution behavior of Chinese university students has the following characteristics: a tendency to construct knowledge, excessive competition, the psychological state of anxiety, and the choice of a single occupation. Fan and Xin (2021) submitted that students participated passively in involution behavior. In considering environmental causes and peer pressure, academic involution behavior also has the connotation of pseudo learning logic and involuntary learning behavior. Based on the above, we submit that academic involution behavior is more appropriately expressed as a behavior mode of increasing burden, excessive competition, and inefficient learning adopted by university students in the face of an uncertain and irrationally competitive environment.

However, the COVID-19 pandemic has had a great impact on the psychology and feelings of university students whose mental health has been affected by long-term social isolation, anxiety, stress, and pessimistic economic predictions. Simultaneously, the increasing trend of psychological exhaustion has promoted a change in people’s attitudes and values (Ahsan, 2014). It is submitted that in such an uncertain environment, university students’ academic involution behavior will become increasingly fierce and the consequences of same will be profound. In the already over competitive learning environment, students will not be able to gain rich life experience or enjoy spiritual growth. However, research on the impact of anxiety and cognitive closure needs on academic involution behavior during the COVID-19 pandemic is scarce. Therefore, this study aims: first, to investigate whether anxiety affects university students’ academic involution behavior during the COVID-19 pandemic, and if so, to what extent; second, to explore the influence path of anxiety on students’ involution behavior based on the mediating effect of cognitive closure needs; and third, to investigate how university students can disengage from involution and establish a more reasonable learning viewpoint.

## Literature review and research hypotheses

### Anxiety and academic involution behavior

The COVID-19 pandemic could had resulted in uncertainty in the society over the economy (Chiang, 2022), employment (Lee and Yang, 2022), academic (Clabaugh et al., 2021), relationships (Elmer et al., 2020), and physical and mental health (Bavolar et al., 2021). Studies have confirmed widespread spikes in the incidence of stress, anxiety, and depression caused by the uncertainty during the COVID-19 pandemic (Daly et al., 2020; Huang and Zhao, 2020; Breslau et al., 2021; O’Connor et al., 2021). Among the general public, younger adults with pre-existing psychological conditions have proven to be at relatively higher risk for related mental health problems (Palgi et al., 2020; Rossi et al., 2020). For university students, the increased social isolation caused by the pandemic, the low efficiency of online teaching, the increased unemployment risk, and uncertainty regarding the future have all exacerbated their anxiety and increased their levels of stress (Durodi’e, 2020; Zheng et al., 2022). At the same time, with the increased risk of being infected with COVID-19, the expanding psychological exhaustion will also promote people’s concept, attitude, and value orientation to change (Ahsan, 2014).

Emotional change plays a decisive role in the subsequent specific actions of individuals. When levels of anxiety and stress increase significantly, it will have a notable impact on an individual’s behavior (Zhang et al., 2016). Moderate anxiety can improve an individual’s alertness and increase an individual’s work and learning capabilities (Wang and Wang, 2007). However, certain studies have indicated that excessive anxiety and stress are not conducive to individuals being able to actively engage in various tasks, resulting in extreme and irrational behaviors, such as aggression and mobile phone addiction (Chiu, 2014; Zhang and Lan, 2020). Research regarding the relationship between anxiety, perceived stress, and academic behavior found that perceived stress can significantly predict academic burnout (Yu, 2017). When students feel that academic pressure and levels of anxiety increase, it leads to a rise in the level of academic burnout (Slivar, 2001; Sevari and Kandy, 2019). In addition, perceived stress and anxiety are significant influencing factors of academic involution.

The general anxiety caused by the COVID-19 pandemic, the reduction of employment opportunities, and the uncertainty of future development prospects have intensified the internal competition among university students, making academic involution behavior prominent among university students (Zheng et al., 2022). Therefore, we propose the first group of hypotheses as follows:

*Hypothesis 1a*: The increased perceived risks associated with the COVID-19 pandemic will significantly increase the anxiety and perceived stress of university students.

*Hypothesis 1b*: Anxiety and perceived stress have a significant positive impact on academic involution behavior.

### Mediating effect of cognitive closure needs

In an environment of general anxiety and uncertainty, individual susceptibility was further moderated by situational variables and individual characteristics (White, 2021). Under the same social conditions, an individual’s distinctive characteristics may affect their ability of coping with uncertainty (Webster and Kruglanski, 1994). For those who are unable or unwilling to tolerate uncertainty, unpredictable factors will increase the negative effects on mental health caused by the COVID-19 pandemic (Berenbaum et al., 2008). Similarly, individuals with an increased need for cognitive closure may make undesirable and irrational decisions under unpredictable circumstances (Berenbaum et al., 2008). Therefore, under unpredictable circumstances, especially the increased risk of uncertainty caused by the COVID-19 pandemic, individuals with high cognitive closure needs may experience greater distress (Chirumbolo and Areni, 2010). For individuals with a relatively high need for cognitive closure, the COVID-19 pandemic may have a stronger effect on their mental health.

During the COVID-19 pandemic, university students may experience a significant increase in levels of stress and anxiety as they face unpredictable situations, such as campus closures, academic delay, and employment uncertainty (Cao et al., 2020; Li et al., 2021). White (2021) predicted that higher cognitive closure needs were associated with higher levels of anxiety and stress during the COVID-19 pandemic. When people with high cognitive closure needs are facing the anxiety and stress caused by uncertainty, their tolerance of uncertainty is extremely low (Rettie and Daniels, 2021). To eliminate the anxiety and stress brought about by this unsettled state, they will try to find certainty and solutions, even if the decisions they make and behaviors they exhibit are irrational (Liu et al., 2007). The anxiety and stress brought about by uncertain situations may cause university students to experience academic involution differently based on their different levels of cognitive closure needs. Based on the above, we propose the following further hypotheses:

*Hypothesis 2a*: The need for cognitive closure plays a mediating role in the relationship between anxiety and academic involution behavior.

*Hypothesis 2b*: For individuals with higher cognitive closure needs, the higher the level of anxiety and stress, the more likely they are to be inclined to academic involution behavior.

## Data collection, assessment tools, and methods

### Data collection

Data were collected from 418 participants, who were university students (including graduate students) from the Inner Mongolia University (Inner Mongolia Autonomous Region), Hebei University (Hebei Province), and Southwest University of Finance and Economics (Sichuan Province) in China. The data were collected through online surveys from May 17, 2022, to June 18, 2022. With the help of Questionnaire Star, an online survey platform, participants visited a website through designated links and provided informed consent to researchers. The participants then completed the COVID-19 perceived risk scale, perceived stress scale (PSS-10), generalized anxiety disorder scale (GAD-7), brief need for closure scale (NFCS-15), and academic involvement behavior scale (AIB-15). After the participants had completed the surveys, the researchers paid them 20 Yuan each and thanked them for their assistance. A total of 402 valid samples (238 female, 164 male; mean age [*M_age_*] = 22.04 years, standard deviation [*SD_age_*] = 2.40; 70 freshman, 77 sophomore, 54 junior, 114 senior, 87 postgraduate) were analyzed after 16 participants’ surveys were excluded due to missing or incomplete responses.

### Assessment tools

#### COVID-19 perceived risk scale

The COVID-19 perceived risk mainly includes two dimensions: people’s cognition and emotion of the adverse effects of COVID-19 (Yldrm and Güler, 2020; Zer et al., 2021). The COVID-19 perceived risk scale consists of two parts. In the first part, participants were asked if they felt that they were at risk for contracting COVID-19 and we assessed the level of perceived risk of COVID-19 infection. In the second part, the participants were asked if they would experience anxiety and fear if they contracted COVID-19, so we could evaluate the fear and anxiety associated with the adverse effects of COVID-19. The first part was composed of three questions and the second part comprised 4 questions. Both parts were ranked on a 5-level Richter scale. The higher the score, the higher the perceived risk of contracting COVID-19. In this study, the reliability was found to be KMO = 0.70, and internal consistency was found to be α = 0.67 (Cronbach’s alpha of α).

#### Generalized anxiety disorder scale

“Anxiety disorder include disorders that share features of excessive fear and anxiety and related behavioral disturbances” [American Psychiatric Association (APA), 2013, p. 189]. Anxiety is often associated with muscle tension and alertness, caution, or avoidance behaviors in preparation for future dangers. Among them, the basic feature of generalized anxiety disorder is excessive anxiety and worry (anxious expectation) about many events or activities [American Psychiatric Association (APA), 2013]. In the field of medical research, there are strict diagnostic criteria for generalized anxiety. Generalized anxiexy disorder-7 (GAD-7) compiled by Spitzer et al. (2006) was widely used in clinical and practical research. GAD-7 can be used for self-assessment of anxiety status of subjects, and can observe the changes of anxiety mood of subjects. Therefore, we will use CAD-7 to measure the university’s students’ generalized anxiety (White, 2021). In the GAD-7, participants were asked to rate the frequency of symptoms such as “excessive worry” in the preceding 2 weeks. The score of each item ranged from 0 (no symptoms) to 3 (nearly daily symptoms), and the total score was between 0 and 21. Scores of 10 and above are considered to be in the clinical range of generalized anxiety. The GAD-7 shows high internal consistency in both clinical samples (α = 0.92; Spitzer et al., 2006) and non-clinical samples (α = 0.90; Rettie and Daniels, 2021). In the anxiety research of Chinese university students, GAD-7 scale had good factorial validity and reliability (α = 0.901; Fu et al., 2021). Furthermore, the validity of GAD-7 in assessing anxiety in the Chinese population has been confirmed (Cao et al., 2020). In the present study, KMO was 0.91, and α was 0.90.

#### Perceived stress scale

Perceived stress refers to the way in which an individual perceives and responds to external stimulus events and whether an individual has confidence or belief in the ability to cope with external stress (Cohen and Williamson, 1988). The perceived stress level of college students is closely related to their anxiety and depression. Perceived stress was measured using the 10-item PSS-10 that was first developed and utilized by Roberti et al. (2006). PSS-10 assesses the stress level of subjects by questioning participants in respect of the frequency of stressful events that have occurred in their lives in the previous month. PSS-10 adopts a 5-level Richter scale, in which 4 reverse scoring questions are asked. Scores on the PSS-10 range from 0 to 40. Roberti et al. (2006) reported Cronbach’s alpha of α = 0.89 in one study. PSS-10 scale had good factorial validity and reliability in the Chinese population, and it has been confirmed (KMO = 0.82, α = 0.82; Chen et al., 2021). In the present study, KMO was 0.82, and α was 0.76.

#### Brief need for closure scale

Cognitive closure needs reflect an individual’s preference, which can be understood as a pursuit of certainty. It is the motive force that makes individuals eager to achieve the goal of closure in processing (Bar-Tal and Kossowska, 2010). Cognitive closure is a continuum, and everyone is in different positions in this continuum. At one end of this continuum, people’s need for cognitive closure is very strong, while at the other end, it is weak. Cognitive closure needs were evaluated using an abbreviated 15-item version of the NFCS-15 developed by Webster and Kruglanski (1994). NFCS-15 includes 5 elements: order, predictability, decisiveness, ambiguity, and close-mindedness (Roets and Van Hiel, 2011). The questionnaire adopts a 6-level Richter scale, which ranges from 1 to 6, and combines the results to produce a composite cognitive closure needs score. The psychometric characteristics and evaluation of the NFCS-15 are similar to those of the longer version of this scale (NFCS-41). The higher the score, the higher the individual’s cognitive need for closure. Roets and Van Hiel (2011) reported that their internal consistency was α = 0.87. The validity and reliability of NFCS-15 in assessing anxiety in the Chinese population have been confirmed (α = 0.80; Liu and Liang, 2007). In this study, KMO was 0.89, and α was 0.84.

#### Academic involvement behavior scale

At present, no AIB-15 has been developed. We developed a 15-item assessment tool to evaluate academic involution behavior based on definitions of the concept by Yang et al. (2022) and Zheng et al. (2022) as well as descriptions of the characteristics of academic involution behavior by Fan and Xin (2021) and Guo (2021). This scale comprises three features: passive learning, inefficient learning, and overlearning. We adopted a 5-level Richter scale and the participants’ answers were given a score of between 1 and 5 points. The scores in respect of all three features were combined to produce a compound score of academic involution behavior. The higher the score, the more severe the level of academic involution behavior. In this study, the reliability was found to be KMO = 0.88, and internal consistency was found to be α = 0.89.

### Variable selection and description

#### Dependent variable

The dependent variable was academic involution behavior, which was measured by the AIB-15. The higher the score, the more serious the level of academic involution behavior encountered. Academic involution includes three features: involuntary learning, inefficient learning, and overlearning.

#### Independent variables

The independent variables included the perceived risk of being infected with COVID-19, anxiety, and perceived stress, which were measured by the relevant scale and were treated as continuous variables. In the mediating effect test, anxiety and perceived stress were combined to generate a new variable: perceived emotion.

#### Mediating variables

The mediating variable was the need for cognitive closure, which was evaluated on a continuum. At one end of the continuum, people’s need for cognitive closure is very strong, while at the other end, it is weak. The higher the score, the higher the need for cognitive closure. The mediating variable was treated as a continuous variable.

#### Control variables

Other control variables that may affect academic involution behavior were also included, such as gender in which male is the reference group; grade, which was divided into graduation grade and non-graduation grade (reference group); career goals, which were divided into clear career goals (reference group) and unclear career goals based on individuals’ subjective evaluation; and campus management, which was divided into closed management (reference group) and unclosed management according to whether the school was open and the form of campus management. Control variables were processed as per the classification variables in models. The descriptive statistical analysis of each variable is shown in Table 1.

**Table 1:** Descriptive statistical analysis of variables.

Variables	Minimum	Maximum	Mean	SD	
Dependent variables	Academic involution	15	75	40.539	10.191
Independent variables	COVID-19 Perceived risk	8	26	18.674	2.723
	Anxiety	0	21	5.488	3.911
	Perceived stress	4	35	16.575	4.853
	Perceived emotion	5	52	22.062	7.765
Mediating variables	Cognitive closure needs	15	82	57.872	10.395
Control variables	Gender	0	1	0.592	0.294
	Grade	0	1	0.500	0.501
	Career goals	0	1	0.338	0.473
	Campus management	0	1	0.472	0.499

### Methods

In this study, we focused on the effects of anxiety and perceived stress on university students’ academic involution behavior and investigated the mediating effect of cognitive closure needs on academic involution behavior. Therefore, we utilized the mediating effect model. To express the influence path of anxiety on academic involution behavior, we also carried out PA-LV utilizing the structural equation model.

#### Mediating effect model

Making use of the studies of Hayes and Andrew (2009) and Wang et al. (2015) for reference, we constructed the following recursive equation for testing the mediating effect:


(1) $$\begin{array}{l} AInvolution_{i}=\alpha_{1}+\beta_{1}\cdot PRisk_{i}+\beta_{2}\cdot PEmotion_{i}\\ \;\;+\beta_{3}X_{i}+\varepsilon_{i} \end{array}$$

(2) $$\begin{array}{l} NCClosure_{i}=\alpha_{2}+\phi {}_{1}\cdot PRisk_{i}+\phi \cdot 2PEmotion_{i}\\ +\mu \cdot X_{i}+\varepsilon_{i} \end{array}$$

(3) $$\begin{array}{l} AInvolution_{i}=\alpha_{3}+\theta_{1}\cdot PRisk_{i}+\theta_{2}\cdot PEmotion_{i}\\ \;+\delta_{1}\cdot NCClosure_{i}+\gamma \cdot X_{i}+\varepsilon_{i} \end{array}$$

where, i represents each participant. The coefficients $\beta_{1}$ , $\beta_{2}$ , and $\beta_{3}$ of Equation (1) are the total effect of the two independent variables of perceived risk and perceived emotion and the control variable on the dependent variable (academic involution). Equation (1) is also a traditional OLS regression equation. The coefficients $\phi_{1},\;\;\phi_{2}$ , and μ of Equation (2) are the effects of independent variables and control variables on the mediating variables (cognitive closure needs), respectively. Coefficient $\delta_{1}$ of Equation (3) is the effect of mediating variable (cognitive closure needs) on the dependent variable (academic involution) after controlling the influence of independent variables (including perceived emotion and perceived risk) and control variables. Coefficients $\theta_{1},\theta_{2}$ , and $\gamma_{1}$ are the direct effects of the independent variables and the control variables on the dependent variables after controlling for the influence of mediating variables.

Intuitively, the independent variable and the control variable change by 1 unit, which will affect the dependent variable changing $\left(\theta_{1}+\theta_{2}\right)AInvolution$ unit through direct effect, and affect the mediating variable changing $\left(\phi {\;}_{1}+\phi_{2}\right)\cdot NCClosure$ units through indirect effect, and the then affect dependent variable changing $\left(\phi {\;}_{1}+\phi_{2}\right)\cdot \delta_{1}\cdot AInvolution$ units through the mediating variable (cognitive closure needs). In other words, the total effect on the dependent variable is $\left(\theta_{1}+\theta_{2}\right)$ plus $\left(\phi {\;}_{1}+\phi_{2}\right)\cdot \delta_{1}$ units when the independent variable changes by 1 unit. Based on the goodness of fit of the model, the Bootstrap sampling test method was used to test the mediating effect, and the sample size was set at 2,000.

#### Path analysis with latent variable model

Although the mediating effect model can identify the role of mediating variables, the specific action path and mechanism of mediating variables on dependent variables is not clear. In addition, it is difficult to identify the influence of independent variables including different indicators (such as cognitive closure needs) on dependent variables through the mediating effect model. With the help of PA-LV, we can estimate the measurement model composed of latent variables and their indicators and analyze the path between variables (i.e., mediating effect test). We can analyze the size, direction, and path of the mediating effect of cognitive closure needs on academic involution behavior more clearly by using PA-LV. It can also be used as a robustness test of the above conclusions.

**Figure 1:**
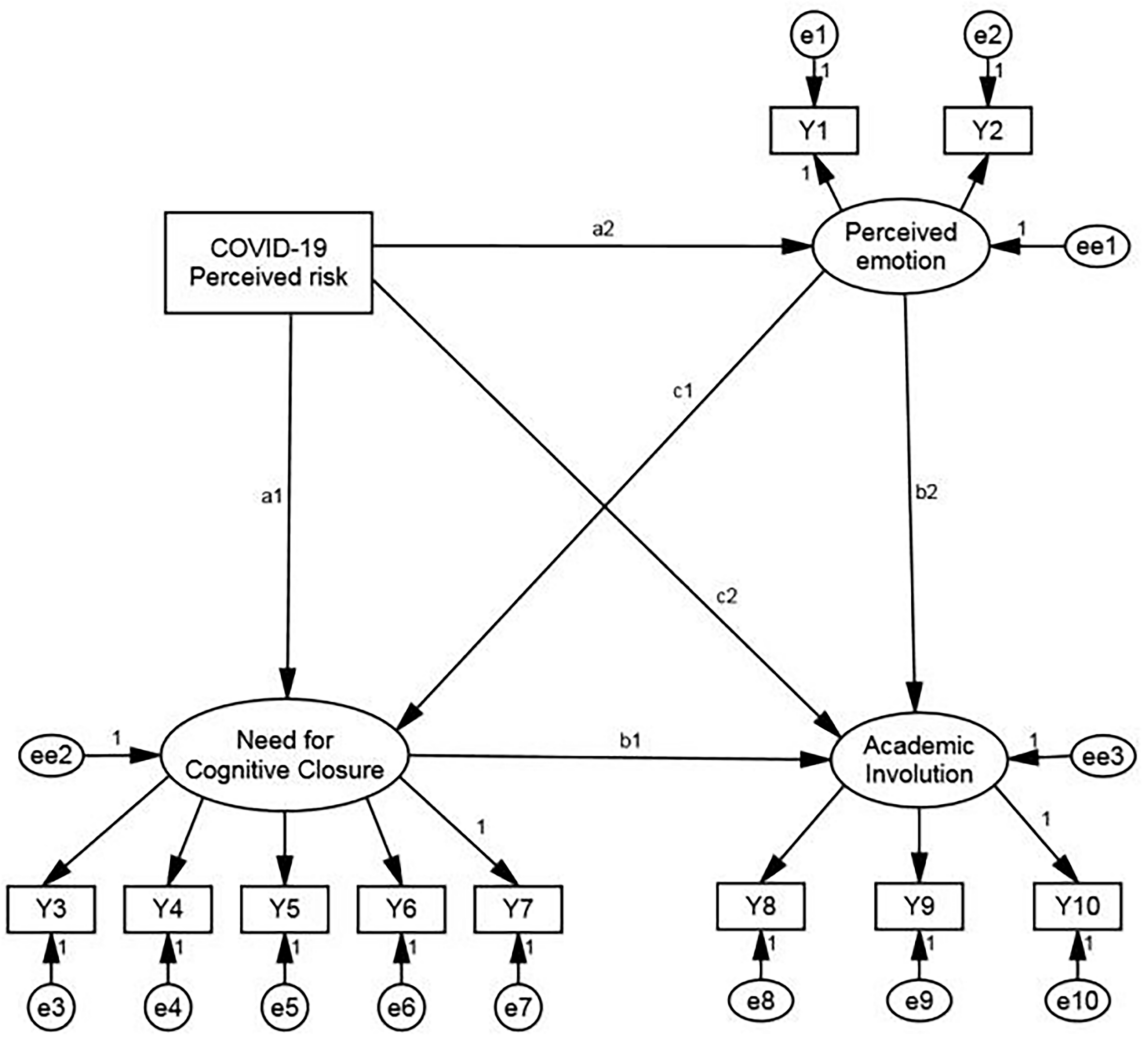
Path analysis with latent variable model.

During the COVID-19 pandemic, every individual is at risk of infection, which, as an objective reality, will affect the emotions of every university student. Therefore, we consider the perceived risk of contracting COVID-19 as an external variable. This was composed of the scores from seven questions. Perceived emotion, cognitive closure needs, and academic involution were all endogenous latent variables. Perceived emotion included two dimensions: anxiety (Y1) and perceived stress (Y2). Cognitive closure needs included five features: order (Y3), predictability (Y4), decisiveness, (Y5), ambiguity (Y6), and close-mindedness (Y7). Academic involution included three indicators: passive learning (Y8), inefficient learning (Y9), and overlearning (Y10). The PA-LV is shown in Figure 1.

## Results

### Factors influencing academic involution behavior

If we accept academic involution as the dependent variable, a regression model can be constructed in which Model 1 only considers the influence of independent variables and Model 2 includes control variables. Models 1 and 2 are multiple linear regression models and Model 3 considers the mediating effect of cognitive closure needs, which is the mediating effect model. The results are shown in Table 2.

**Table 2:** Regression results of factors influencing academic involution behavior.

Variables	Model 1	Model 2	Model 3
Intercept	23.380^***^(3.574)	26.588^***^(3.635)	16.826^***^(3.652)
Dependent variables			
COVID-19 Perceived risk	0.411^**^(0.184)	0.321^*^(0.183)	0.075 (0.180)
Anxiety	0.356^**^(0.151)	0.408^***^(0.149)	
Perceived stress	0.454^**^(0.119)	0.398^***^(0.117)	
Perceived emotion			0.362^***^(0.061)
Control variable			
Gender (female)		2.575^**^(1.016)	1.425 (0.995)
Grade (graduation grade)		−0.964 (0.967)	−0.937 (0.927)
Career goals (clear)		−2.174^**^(0.995)	−1.907 (0.962)
Campus management (unclosed)		−2.507^***^(0.948)	−2.466^***^(0.910)
Mediating variables			
Cognitive closure needs			0.270^**^(0.047)
Adj-R2	0.130	0.173	
*F*-value	*F*(3,398) = 19.88^***^	*F*(7,394) = 11.79^***^	*F*(7,294) = 17.467

*p < 0.1; **p < 0.05; ***p < 0.01. The number in brackets is the standard error.

The results of Models 1 and 2 indicate that irrespective of whether mediating variables are considered, the effects of anxiety and perceived stress on academic involution behavior are positive and significant. Considering the results of Model 2, we see that the score of academic involution behavior increases by 0.408 (*p* < 0.01) and 0.398 (*p* < 0.01) units for each increase of the anxiety and perceived stress scores. This demonstrates that the anxiety and perceived stress associated with the COVID-19 pandemic has aggravated academic involution behavior and Hypothesis 1b has been verified. The sudden social crisis caused by the COVID-19 pandemic has had an impact on the psychology of university students.

We found that females were more likely to experience academic involution than males, and their academic involution scores were on average 2.575 units higher than males (*p* < 0.05). Considering career goals, the findings suggested that individuals with unclear career goals had a 2.174 (*p* < 0.05) lower academic involution score than those individuals with clear career goals. When we considered the aspect of campus management and compared with individuals under closed and open campus management, we see that the academic involution behavior scores of individuals under unclosed management campuses were an average of 2.057 units lower (*p* < 0.01). This is probably due to the fact that in a closed management environment, students’ anxiety will increase significantly.

The anxiety, stress, and uncertainty caused by the COVID-19 pandemic have a common impact on universities. However, an individual’s characteristics and the different situations that they find themselves in will further modify an individual’s perception of the anxiety and stress. Differing levels of the need for cognitive closure will make each individual feel differently about anxiety, stress, and uncertainty. Model 3 tested the mediating effect of cognitive closure needs between perceived emotion (including anxiety and perceived stress) and academic involution, as well as the perceived risk of contracting COVID-19 and academic involution. The regression coefficient of mediating effect was 0.270 (*p* < 0.01), which is positive and significant. This indicates that the perceived risk of contracting COVID-19, anxiety, and perceived pressure not only directly affect university students’ academic involution behavior but also indirectly affect students’ academic involution behavior through the mediating path of cognitive closure needs. Research Hypothesis 2a was verified.

### Mediating effect test of cognitive closure needs

Model 3 in Table 2 is a mediating effect model constructed with the perceived risk of contracting COVID-19 and perceived emotion as independent variables, academic involution behavior as the dependent variable, and cognitive closure needs as the mediating variable. Our results indicate that the mediating effect exists and is significant. Table 3 provides more information in respect of the analysis process and results of the mediating effect testing.

**Table 3:** Mediating effect results and tests.

Results	Total effects $\left(\phi {\;}_{1}+\phi_{2}\right)\cdot \delta_{1}+\left(\theta_{1}+\theta_{2}\right)$	Direct effects $\left(\theta_{1}+\theta_{2}\right)$	Mediating effects $\left(\phi {\;}_{1}+\phi {\;}_{2}\right)\cdot \delta_{1}$	Ratio (%)
COVID-19 perceived risk⟹ Cognitive closure needs⟹ Academic involution	0.322	0.075	0.246	100
Perceived emotion⟹ Cognitive closure needs⟹ Academic involution	0.402***	0.362**	0.014	10.150
Tests	Tests results	Mediating effects (Z value)	Mediating effects (*P* value)	(95% Boot CI)
COVID-19 perceived risk⟹ Cognitive closure needs⟹ Academic involution	Complete mediating effect	11.320	0.000	0.023 ~ 0.113
Perceived emotion⟹ Cognitive closure needs⟹ Academic involution	Partial mediating effect	2.712	0.007	0.005 ~ 0.063

**p < 0.05; ***p < 0.01.

The analysis of the mediating effect results shows that cognitive closure needs have a complete mediating effect on the relationship between the perceived risk of contracting COVID-19 and academic involution, with a value of 0.246 (*p* < 0.00). However, cognitive closure needs have a partial mediating effect on the relationship between perceived emotion and academic involution, with a value of 0.014 (*p* < 0.01), accounting for 10.15% of the total effect. Using Bootstrap sampling inspection method, the same results are obtained. This indicates that the perceived risk of contracting COVID-19 affects individuals’ academic involution behavior entirely through the channel of cognitive closure needs. While perceived emotion not only indirectly impacts individuals’ academic involution behavior through the mediating variable (cognitive closure needs), it also directly affects individuals’ academic involution behavior. This also further verified Hypothesis 2a.

### Path analysis with latent variable

**Figure 2:**
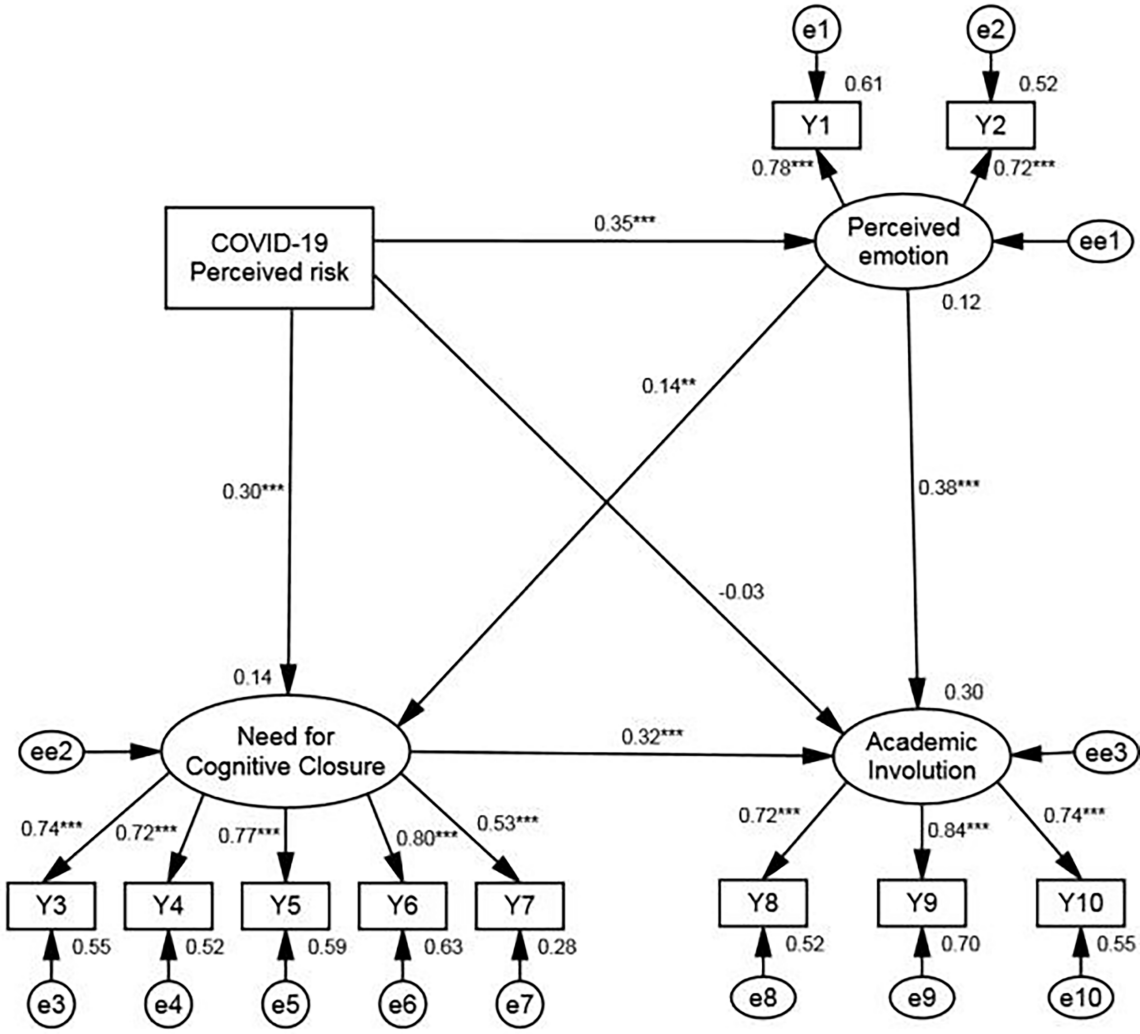
Results of path analysis with latent variable.

According to the constructed PA-LV model, we calculated the model using Amos26.0 software, and the results indicated that: $\chi^{2}/df=3.756,$ although not less than 3, but between 0 and 5, the result is acceptable. GFI=0.931, AGFI=0.897, IFI=0.932, TLI=0.903, and CFI=0.931, their values are greater than or close to 0.90. The RMSEA index is 0.083, which is near 0.08, indicating that the model fitness is relatively good. The results are shown in Figure 2 and Table 4.

**Table 4:** Mediating effect results and tests of path analysis with latent variable.

Mediating effects	Coefficient	Lower	Upper	*p*-Value
COVID-19 perceived risk⟹ Cognitive closure needs ⟹ Academic involution	0.106	0.047	0.185	0.001
COVID-19 perceived risk⟹ Perceived emotion ⟹ Academic involution	0.149	0.090	0.242	0.001
Perceived emotion ⟹ Cognitive closure needs ⟹ Academic involution	0.044	0.005	0.103	0.030
COVID-19 perceived risk⟹ Perceived emotion ⟹ Cognitive closure needs⟹ Academic involution	0.017	0.003	0.043	0.024
All mediating effects	0.317	0.212	0.444	0.001

In Figure 2, we see that except for the direct effect of COVID-19 perceived risk on academic involution being insignificant, the other path coefficients are significant. This is consistent with the mediating effect test of the need for cognitive closure. Cognitive closure needs provide a complete mediating effect in COVID-19 perceived risk and academic degradation, so the direct effect is 0, which demonstrates that in the PA-LV model, the path coefficient from COVID-19 perceived risk to academic involution is insignificant.

In Table 4, the results of PA-LV, the mediating effect of cognitive closure needs in the relationship between COVID-19 perceived risk and academic involution, are shown more clearly as well as in the relationship between perceived emotions (including anxiety and perceived stress) and academic involution. Compared with the results displayed in Table 3, the PA-LV model also indicates the path analysis results of the chain mediating effect of COVID-19 perceived risk on perceived emotion to cognitive closure needs to academic involution.

Figure 2 shows that the path coefficient from the perceived risk of contracting COVID-19 to perceived emotion is 0.352 (*p* < 0.01), which indicates that during the COVID-19 pandemic, the increase in the perceived risk of contracting COVID-19 has indeed led to a rise in the anxiety and stress levels of university students. Therefore, Hypothesis 1a is verified. From the results of the mediating effect produced by cognitive closure needs, we see that: (1) the mediating effect value of COVID-19 perceived risk to cognitive closure needs to academic involution was 0.106 (*p* < 0.01), (2) the mediating effect value of perceived emotion to cognitive closure needs to academic involution was 0.044 (*p* < 0.05), and (3) the chain mediating effect value of COVID-19 perceived risk to perceived emotion to cognitive closure needs to academic involution was 0.017 (*p* < 0.05). The mediating effects caused by cognitive closure needs are positive and significant, and Hypothesis 2b was basically verified. It is, therefore, submitted that individuals with higher cognitive closure needs are more likely to display academic involution behavior when they experience higher levels of anxiety and perceived pressure.

## Conclusion and discussion

### Conclusion

The main purpose of our study was to evaluate the impact of anxiety and perceived stress on university students’ academic involution behavior during the COVID-19 pandemic, and to evaluate cognitive closure needs as a mediating variable between perceived emotion (including anxiety and stress) and academic involution behavior. We analyzed the survey data of 402 university students from three universities in China for empirical analysis, and the results support our all hypothesis.

First, university students with high COVID-19 perceived risk experienced higher levels of anxiety and stress, which directly affected their academic involution behavior it also had a mediating effect through the cognitive closure needs of different individuals. University students’ perceived risk of contracting COVID-19 affected their academic involution behavior through cognitive closure needs, which had a complete mediating effect between COVID-19 perceived risk and academic involution behavior. At the same time, cognitive closure needs had a partial mediating effect on the relationship between perceived emotion and academic involution behavior.

In addition, among other factors affecting academic involution behavior, we found that females were more likely to experience academic involution than males. University students with clear career goals are more likely to experience academic involution behavior. We submit that closed campus management creates a relatively repressive and highly competitive atmosphere among students. As a result, compared with the campus without closed management, university students on closed management campus are more likely to fall into academic involution. At the same time, college students of different grades did not show significant differences in academic involution behavior.

## Discussion

For profound reasons, the COVID-19 pandemic has made the risk associated with life increasingly prominent. Isolation and the suspension of education and business activities caused by the COVID-19 pandemic have had a direct impact on every individual and organization (Gong, 2020). Unpredictable academic and employment situations are feared for the future (Li et al., 2020; Karakose, 2021). Individuals are not only afraid of contracting COVID-19 but also fear the stagnation of life and reduction of opportunities caused by the pandemic (Chandran et al., 2020). University students, who are in a transitional stage of their lives, are experiencing inner turmoil. This is a stage of vacillation and those who want to obtain a sense of security and stability, that is, individuals with high cognitive closure needs may be more likely to engage in academic involution behavior.

Therefore, we submit that the COVID-19 pandemic, which has intensified the likelihood of exposure to danger and increased uncertainty in modern society, has forced university students to exhibit compulsory autonomy, driving them to become active and independent individuals. It is precisely their extreme enthusiasm and autonomy that reflects their internal fear and anxiety (Dhar et al., 2020). For example, the fact that the worth of a diploma is relatively devalued is a constant reminder to students of the risk of unemployment, which has increased during the COVID-19 pandemic (Shen et al., 2021). When they do not know how to make the correct decision, they will choose not to give up on the possibility of academic success. The uncertainty created by the COVID-19 pandemic has resulted in irrational, inefficient, and overly competitive academic involution behavior.

The COVID-19 pandemic has been, and continues to be, a period in human history that is marked by uncertainty. University students are facing an unpredictable future. Although the uncertainty of the COVID-19 era seems unprecedented, we must remember that life, in general, has been, and will always be, unpredictable (White, 2021). For many students, the pandemic has been an opportunity to enhance their abilities through different learning methods and technologies. Firstly university students should recognize that the academic involution behavior originates from the confusion of the present and future. In the increasingly fierce competition, especially under the influence of the pandemic, students’ lack of experience leads to their inability to get out of the homogeneous and integrated competition (Zheng et al., 2022). Therefore, universities should strengthen mental health education and improve the psychological quality of university students. Universities should help students improve their self-awareness in the process of cultivating them and improve their ability to face setbacks and cope with uncertain risks. University students need to be able to objectively understand, evaluate, analyze and reflect on themselves, so that they can clearly understand their advantages and disadvantages. Moreover, University should learn to set appropriate goals according to our own actual situation, so as to avoid blindly following the trend.

Secondly, university students should establish correct employment values (Li et al., 2020). With the severe employment situation caused by COVID-19 pandemic in the whole society, the employment situation of college graduates is not optimistic. Therefore, universities should organize regular psychological assessments on university students’ academic and employment anxiety, so as to timely understand and grasp the psychological status and behavioral changes of university students during the COVID-19 pandemic. Students found to have psychological problems should be given psychological counseling in time, and those with serious problems should be given psychological crisis intervention in time, and regular follow-up should be carried out. Through these measures, university students can be helped to adjust their mentality, so as to reduce the psychological pain caused by uncertainty and avoid irrational academic involution.

## Data availability statement

The original contributions presented in the study are included in the article/Supplementary material, further inquiries can be directed to the corresponding author.

## Author contributions

DY and SG conceived of the presented idea. DY and HZ designed the methods of data collection and performed the data analysis. DY, SG, and HZ wrote the first draft of the manuscript. WZ wrote and edited the manuscript. All authors contributed to the article and approved the submitted version.

## Conflict of interest

The authors declare that the research was conducted in the absence of any commercial or financial relationships that could be construed as a potential conflict of interest.

## Publisher’s note

All claims expressed in this article are solely those of the authors and do not necessarily represent those of their affiliated organizations, or those of the publisher, the editors and the reviewers. Any product that may be evaluated in this article, or claim that may be made by its manufacturer, is not guaranteed or endorsed by the publisher.

## Supplementary material

The Supplementary material for this article can be found online at: https://www.frontiersin.org/articles/10.3389/fpsyg.2022.1005708/full#supplementary-material
