# Supplementary material for: Influence of anxiety on university students’ academic involution behavior during COVID-19 pandemic: Mediating effect of cognitive closure needs
Source: Front Psychol. 2022 Sep 29;13:1005708. doi: 10.3389/fpsyg.2022.1005708 (PMC9558283; doi:10.3389/fpsyg.2022.1005708)
Supplement: Supplementary file 1 [file Table_1.docx]

**Influence of Anxiety on University Students’ Academic Involution Behavior During COVID-19 Pandemic**

**Part I COVID-19 perceived risk scale**

| Questions | Please answer the following questions according to your true feelings | Strongly Disagree | Moderately Disagree | Slightly Agree | Moderately Agree | Strongly Agree |
| --- | --- | --- | --- | --- | --- | --- |
| A1. | I think I may be infected with COVID-19. | 1 | 2 | 3 | 4 | 5 |
| A2. | I often worry about to much my physical condition because of COVID-19. | 1 | 2 | 3 | 4 | 5 |
| A3. | I think my risk of infecting COVID-19 is increasing | 1 | 2 | 3 | 4 | 5 |
| A4. | I have felt anxiety, fear and tension because of the COVID-19. | 1 | 2 | 3 | 4 | 5 |
| A5. | COVID-19 has aggravated my fear and anxiety about the uncertainty of the future | 1 | 2 | 3 | 4 | 5 |
| A6. | I will feel anxious and uneasy about my future employment because of the COVID-19 | 1 | 2 | 3 | 4 | 5 |
| A7. | I think the impact of COVID-19 on me is negligible. | 1 | 2 | 3 | 4 | 5 |

**Part II Generalized anxiety disorder scale**

| Questions | Over the last 2 weeks, how often have you been bothered by the following problems? | Not at all | Several days | More than half  the days | Nearly every  day |
| --- | --- | --- | --- | --- | --- |
| B1. | Feeling nervous, anxious or on edge | 0 | 1 | 2 | 3 |
| B2. | Not being able to stop or control worrying | 0 | 1 | 2 | 3 |
| B3. | Worrying too much about different things | 0 | 1 | 2 | 3 |
| B4. | Trouble relaxing | 0 | 1 | 2 | 3 |
| B5. | Being so restless that it is hard to sit still | 0 | 1 | 2 | 3 |
| B6. | Becoming easily annoyed or irritable | 0 | 1 | 2 | 3 |
| B7. | Feeling afraid as if something awful might happen | 0 | 1 | 2 | 3 |

**Part III Perceived stress scale**

| Questions | The questions in this scale ask you about your feelings and thoughts during the last mouth.in each case, you will be asked yo indicate by circling how often you felt or thought a certain way. | Never | Almost never | Sometimes | Fairly Often | Very Often |
| --- | --- | --- | --- | --- | --- | --- |
| C1. | In the last month, how often have you been upset because of something that happened unexpectedly? | 0 | 1 | 2 | 3 | 4 |
| C2. | In the last month, how often have you felt that you were unable to control the important things in your life? | 0 | 1 | 2 | 3 | 4 |
| C3. | In the last month, how often have you felt nervous and "stressed"? | 0 | 1 | 2 | 3 | 4 |
| C4. | In the last month, how often have you felt confident about your ability to handle your personal problems? | 0 | 1 | 2 | 3 | 4 |
| C5. | In the last month, how often have you felt that things were going your way? | 0 | 1 | 2 | 3 | 4 |
| C6. | In the last month, how often have you found that you could not cope with all the things that you had to do? | 0 | 1 | 2 | 3 | 4 |
| C7. | In the last month, how often have you been able to control irritations in your life? | 0 | 1 | 2 | 3 | 4 |
| C8. | In the last month, how often have you felt that you were on top of things? | 0 | 1 | 2 | 3 | 4 |
| C9. | In the last month, how often have you been angered because of things that happened that were outside of your control? | 0 | 1 | 2 | 3 | 4 |
| C10 | In the last month, how often have you felt difficulties were piling up so high that you could not overcome them? | 0 | 1 | 2 | 3 | 4 |

**Part IV Brief need for closure scale**

| Questions | Read each of the following statements and decide how much you agree with each according to your beliefs and experiences. Please respond according to the following scale | Strongly Disagree | Moderately Disagree | Slightly Disagree | Slightly Agree | Moderately Agree | Strongly Agree |
| --- | --- | --- | --- | --- | --- | --- | --- |
| D1. | I don’t like situations that are uncertain. | 1 | 2 | 3 | 4 | 5 | 6 |
| D2. | I dislike questions which could be answered in many different ways. | 1 | 2 | 3 | 4 | 5 | 6 |
| D3. | I find that a well ordered life with regular hours suits my temperament. | 1 | 2 | 3 | 4 | 5 | 6 |
| D4. | I feel uncomfortable when I don’t understand the reason why an event occurred in my life. | 1 | 2 | 3 | 4 | 5 | 6 |
| D5. | I feel irritated when one person disagrees with what everyone else in a group believes. | 1 | 2 | 3 | 4 | 5 | 6 |
| D6. | I don’t like to go into a situation without knowing what I can expect from it. | 1 | 2 | 3 | 4 | 5 | 6 |
| D7. | When I have made a decision, I feel relieved. | 1 | 2 | 3 | 4 | 5 | 6 |
| D8. | When I am confronted with a problem, I’m dying to reach a solution very quickly. | 1 | 2 | 3 | 4 | 5 | 6 |
| D9. | I would quickly become impatient and irritated if I would not find a solution to a problem immediately | 1 | 2 | 3 | 4 | 5 | 6 |
| D10 | I don’t like to be with people who are capable of unexpected actions. | 1 | 2 | 3 | 4 | 5 | 6 |
| D11. | I dislike it when a person’s statement could mean many different things. | 1 | 2 | 3 | 4 | 5 | 6 |
| D12. | I find that establishing a consistent routine enables me to enjoy life more. | 1 | 2 | 3 | 4 | 5 | 6 |
| D13. | I enjoy having a clear and structured mode of life. | 1 | 2 | 3 | 4 | 5 | 6 |
| D14. | I do not usually consult many different opinions before forming my own view. | 1 | 2 | 3 | 4 | 5 | 6 |
| D15. | I dislike unpredictable situations. | 1 | 2 | 3 | 4 | 5 | 6 |

**Part V Academic involvement behavior scale**

| Questions | Read each sentence below and decide how much you agree with each sentence according to your learning situation. Please answer in the following proportion. | Strongly Disagree | Moderately Disagree | Slightly Agree | Moderately Agree | Strongly Agree |
| --- | --- | --- | --- | --- | --- | --- |
| E1. | I study anxiously in order to keep up with others' learning rhythm. | 1 | 2 | 3 | 4 | 5 |
| E2. | When I don't do something that most people do,I will feel uneasy | 1 | 2 | 3 | 4 | 5 |
| E3. | I will compare my academic performance ranking with others, forcing myself to devote yourself to learning. | 1 | 2 | 3 | 4 | 5 |
| E4. | There is no clear purpose for obtaining various certificates, just because everyone takes the exam. | 1 | 2 | 3 | 4 | 5 |
| E5. | I am very resistant to the fierce involution competition, but I was forced to participate. | 1 | 2 | 3 | 4 | 5 |
| E6. | To surpass others academically, I will go to the classroom or library early to study, even if the learning efficiency is not high? | 1 | 2 | 3 | 4 | 5 |
| E7. | Because of the academic involution, I keep working hard in your studies, but it is difficult to achieve better results. | 1 | 2 | 3 | 4 | 5 |
| E8. | For the homework and papers assigned by the teacher, I try to get higher scores by simply increasing the number of words. | 1 | 2 | 3 | 4 | 5 |
| E9. | It's easy to feel frustrated in academic,and feel that I have entered a dead cycle of energy consumption. | 1 | 2 | 3 | 4 | 5 |
| E10 | I often feel that my efforts are not rewarded, but I have to continue to work hard. | 1 | 2 | 3 | 4 | 5 |
| E11. | In order to get academic ranking and improve my competitiveness, I join organizations such as the student union and strive for more positions | 1 | 2 | 3 | 4 | 5 |
| E12. | In order to have more resources in the future, I constantly pursue various learning opportunities and participate in various activities. | 1 | 2 | 3 | 4 | 5 |
| E13. | Excessive academic involution brings me physical discomfort, such as headache, irritability and so on. | 1 | 2 | 3 | 4 | 5 |
| E14. | I belong to people who would rather work harder than surpass others | 1 | 2 | 3 | 4 | 5 |
| E15. | For the GPA and scholarship, I hold the attitude of "you work hard, I work harder than you", and blindly compete, leading to my exhaustion. | 1 | 2 | 3 | 4 | 5 |
